# Supplementary material for: Generation of large amplitude phonon states in quantum acoustics
Source: Nat Commun. 2025 Jul 2;16:6096. doi: 10.1038/s41467-025-61237-8 (PMC12223055; doi:10.1038/s41467-025-61237-8)
Supplement: Supplementary file 1 — Supplementary Information [file 41467_2025_61237_MOESM1_ESM.pdf]

# Supplementary Information: Large Amplitude Phonon States in Quantum Acoustics

C.A. Potts[1,†], W.J.M. Franse[1], V.A.S.V. Bittencourt[2], A. Metelmann[2,3,4] and G.A. Steele[1,*]

[1] *Kavli Institute of Nanoscience, Delft University of Technology, PO Box 5046, 2600 GA Delft, The Netherlands*
[2] *ISIS (UMR 7006), Université de Strasbourg, 67000 Strasbourg, France*
[3] *Institute for Theory of Condensed Matter, Karlsruhe Institute of Technology, 76131 Karlsruhe, Germany*
[4] *Institute for Quantum Materials and Technology, Karlsruhe Institute of Technology, 76344 Eggenstein-Leopoldshafen, Germany*

[†]Email: clinton.potts@nbi.ku.dk
[*]Email: g.a.steele@tudelft.nl

## I. SUPPLEMENTARY NOTE 1: THE DEVICE

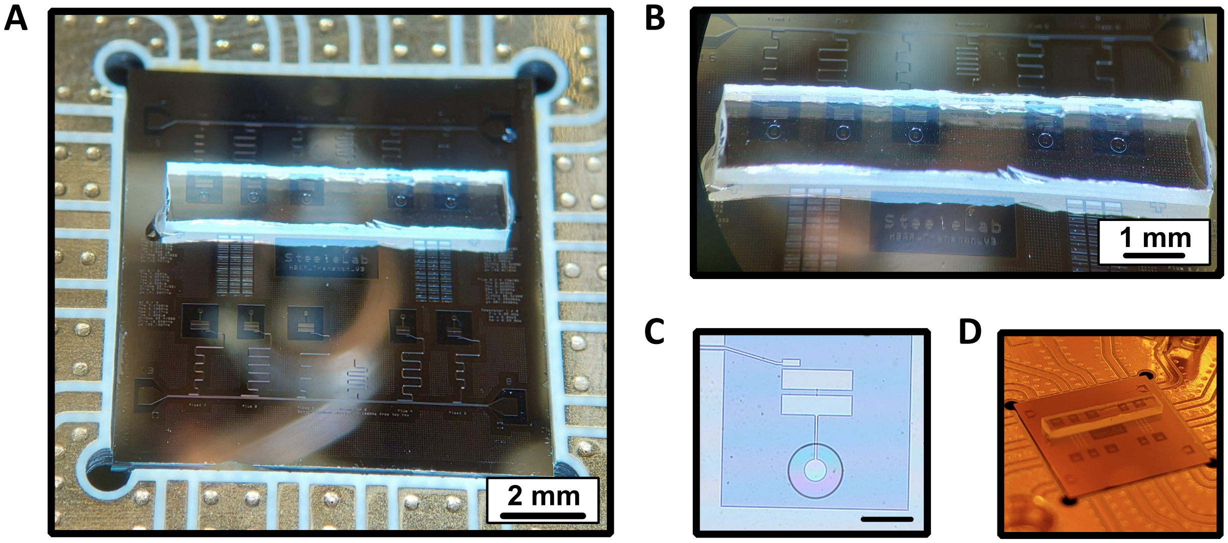


SUPPLEMENTARY FIGURE 1. **Fabricated flip-chip HBAR Device.** (A) Optical micrograph of the assembled flip-chip device. The top feedline of qubits is fabricated with a flip-chip of sapphire, and the bottom feedline has no sapphire flip-chip as references. (B) Zoomed optical micrograph of the flip-chip assembly. (C) Optical micrograph of the qubit with the sapphire chip assembled on top. The overlap between the antenna and the 250$\mu$m aluminum nitride piezoelectric transducer is visible. The scale bar is 250$\mu$m. (D) Optical micrograph of the entire chip loaded in the printed circuit board.

## II. SUPPLEMENTARY NOTE 2: THEORY

### A. System Hamiltonian and Master Equation

The system consists of a microwave cavity coupled to a transmon qubit which in turn is coupled to an HBAR phonon mode. The transmon qubit can be considered, up to a good approximation, to be a two-level system. The readout cavity is driven two with coherent tones: one at a frequency $\omega_p$, which we call the probe tone, and one at a frequency $\omega_d$, which we call the drive tone. The experiment measures the coupling between the qubit and the phonon mode via two-tone spectroscopy performed via the cavity. The procedure consists of considering the cavity detuned from the qubit, setting the probe tone at the (shifted) cavity frequency and varying the drive tone close to the qubit Lamb-shifted frequency. The transmission of the cavity carries information about the qubit correlations $\langle \hat{\sigma}_z \rangle$. The phonon mode has a frequency close to the qubit frequency.

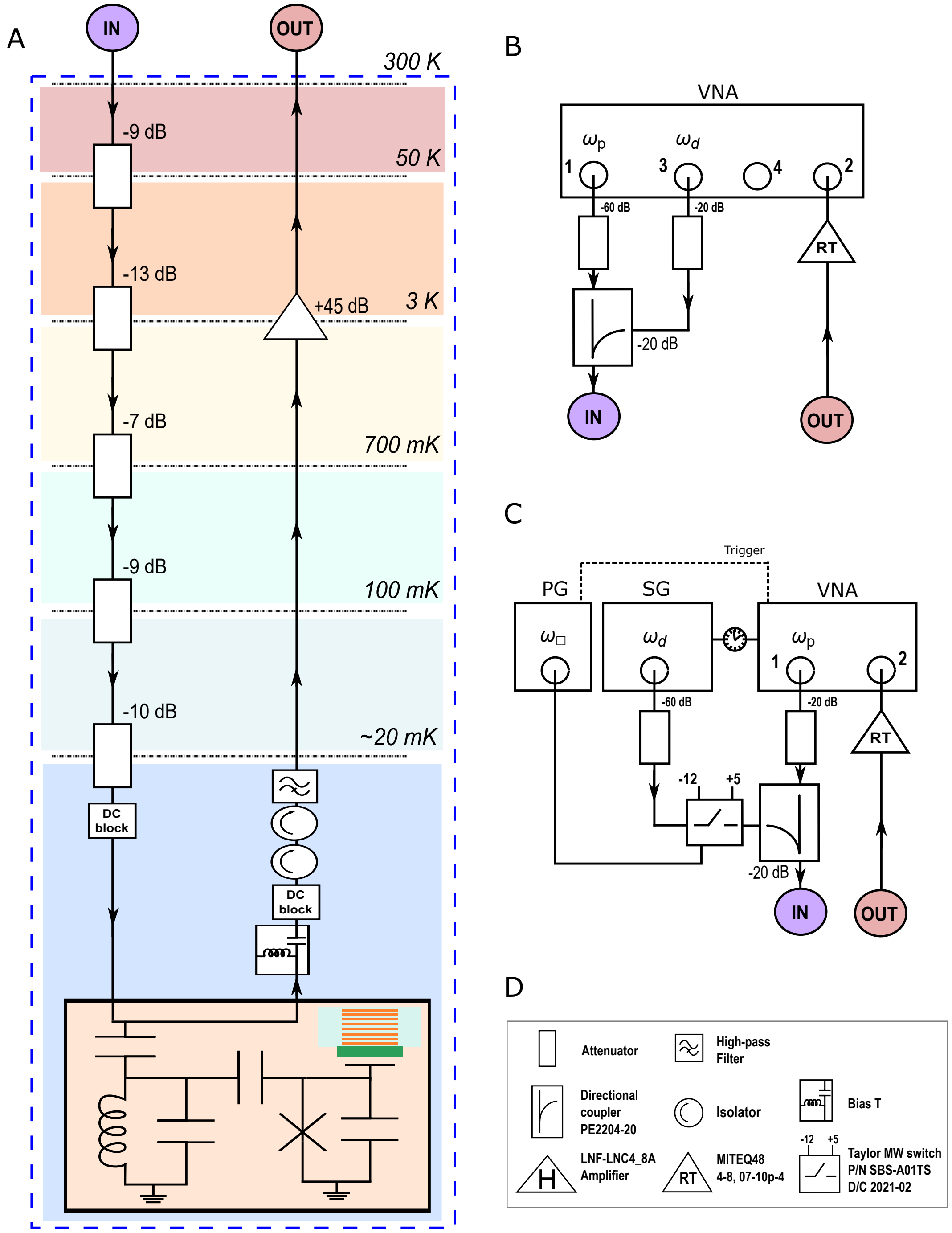


SUPPLEMENTARY FIGURE 2. **Schematic of the measurement setup.** (A) Dilution refrigerator wiring setup. Outside the refrigerator, we used two different setups. In (B), we show our Two-tone spectroscopy configuration used for qubit spectroscopy. Here, we sent a weak continuous wave tone (readout resonator probe) from the vector network analyzer (VNA) port one and a second continuous wave tone (qubit drive) from the VNA port 3. These two signals are combined using a directional coupler before entering the dilution refrigerator. The signal from the dilution refrigerator goes through a room-temperature amplifier before it goes to port 2 of the VNA. In (C), we show our "time domain" setup. Here, we replaced output port 3 of the VNA with a signal generator (SG) to provide the drive tone. A switch (*Taylor MW switch*) together with a pulse generator (PG, *Rigol DG1022*) is placed between the directional coupler and the signal generator. Adapted from[1]

We model the system with the Hamiltonian

$$\begin{aligned}\frac{\hat{\mathcal{H}}}{\hbar} &= \omega_{\rm r}\hat{a}^\dagger\hat{a} + \omega_{\rm b}\hat{b}^\dagger\hat{b} + \frac{\omega_{\rm q}}{2}\hat{\sigma}_{\rm z} + g_{\rm qc}(\hat{a}\hat{\sigma}_+ + \hat{a}^\dagger\hat{\sigma}_-) + g_{\rm qb}(\hat{b}^\dagger\hat{\sigma}_- + \hat{b}\hat{\sigma}_+) \\ &\quad + \epsilon_{\rm d}(\hat{a}e^{i\omega_{\rm d}t} + \hat{a}^\dagger e^{-i\omega_{\rm d}t}) + \epsilon_{\rm p}(\hat{a}e^{i\omega_{\rm p}t} + \hat{a}^\dagger e^{-i\omega_{\rm p}t}),\end{aligned} \tag{S1}$$

where $\hat{a}^{(\dagger)}$ are the annihilation (creation) operators for the readout cavity with frequency $\omega_{\rm r}$, $\hat{b}^{(\dagger)}$ are the annihilation (creation) operators for the HBAR mode with frequency $\omega_{\rm b}$, $\hat{\sigma}_{\rm z}$ is the qubit population operator with qubit frequency $\omega_{\rm q}$. The couplings are defined by the rates $g_{\rm qc}$ between the qubit and the readout cavity and $g_{\rm qb}$ between the qubit and the HBAR mode, where we assume that $g_{\rm qc} \gg g_{\rm qb}$. Finally, the two drives are described by the amplitude $\epsilon_{\rm p,d}$ with frequencies $\omega_{\rm p,d}$. The system is operated in the cavity-qubit dispersive regime $g_{\rm qc} \ll |\omega_{\rm r} - \omega_{\rm q}|$. We then consider the standard Schrieffer-Wolff transformation up to the first order in $g_{\rm qc}/|\omega_{\rm r} - \omega_{\rm q}|$. Defining $\chi = g_{\rm qc}^2/(\omega_{\rm q} - \omega_{\rm r})$, the transformed Hamiltonian reads

$$\begin{aligned}\frac{\hat{\mathcal{H}}'}{\hbar} &= \frac{\tilde{\Omega}_{\rm q}}{2}\hat{\sigma}_{\rm z} + \omega_{\rm r}\hat{a}^\dagger\hat{a} + \omega_{\rm b}\hat{b}^\dagger\hat{b} + \chi\hat{a}^\dagger\hat{a}\hat{\sigma}_{\rm z} + g_{\rm qb}(\hat{b}^\dagger\hat{\sigma}_- + \hat{b}\hat{\sigma}_+) - \frac{g_{\rm qb}}{g_{\rm qc}}\chi(\hat{a}^\dagger\hat{b} + \hat{a}\hat{b}^\dagger)\hat{\sigma}_{\rm z} \\ &\quad + \sum_{j=p,d}\epsilon_{\rm j}(\hat{a}e^{i\omega_j t} + \hat{a}^\dagger e^{-i\omega_j t}) + \frac{g_{\rm qc}}{\omega_{\rm r} - \omega_{\rm q}}\sum_{j=p,d}\epsilon_{\rm j}(\hat{\sigma}_+ e^{i\omega_j t} + \hat{\sigma}_- e^{-i\omega_j t}).\end{aligned} \tag{S2}$$

The Lamb-shifted qubit frequency is $\tilde{\Omega}_{\rm q} = \omega_{\rm q} + \chi$. Given the system's parameters, we will discard the qubit-mediated beam-splitter term $\frac{g_{\rm qb}}{g_{\rm qc}}\chi(\hat{a}^\dagger\hat{b} + \hat{a}\hat{b}^\dagger)\hat{\sigma}_{\rm z}$, as it is several orders of magnitude smaller than $g_{\rm qb}$. Furthermore, in the two-tone spectroscopic setup, we can retain only the probe term for the cavity and only the drive term for the qubit. With such approximations, we have

$$\begin{aligned}\frac{\hat{\mathcal{H}}'}{\hbar} &= \frac{\tilde{\Omega}_{\rm q}}{2}\hat{\sigma}_{\rm z} + \omega_{\rm r}\hat{a}^\dagger\hat{a} + \omega_{\rm b}\hat{b}^\dagger\hat{b} + \chi\hat{a}^\dagger\hat{a}\hat{\sigma}_{\rm z} + g_{\rm qb}(\hat{b}^\dagger\hat{\sigma}_- + \hat{b}\hat{\sigma}_+) \\ &\quad + \epsilon_{\rm p}(\hat{a}e^{i\omega_{\rm p}t} + \hat{a}^\dagger e^{-i\omega_{\rm p}t}) + \varepsilon_{\rm d}(\hat{\sigma}_+ e^{i\omega_{\rm d}t} + \hat{\sigma}_- e^{-i\omega_{\rm d}t}),\end{aligned} \tag{S3}$$

where we have defined $\varepsilon_{\rm d} = \frac{g_{\rm qc}\epsilon_{\rm d}}{\omega_{\rm q} - \omega_{\rm r}}$. It is convenient to move to a frame co-rotating with the pump and the probe frequencies, for which the Hamiltonian in Eq. (S3) reads

$$\begin{aligned}\frac{\hat{\mathcal{H}}'_{\rm rot}}{\hbar} &= -\frac{\Delta_{\rm q}}{2}\hat{\sigma}_{\rm z} + (-\Delta_{\rm r} + \chi)\hat{a}^\dagger\hat{a} - \Delta_{\rm b}\hat{b}^\dagger\hat{b} + \chi\hat{a}^\dagger\hat{a}\hat{\sigma}_{\rm z} \\ &\quad + g_{\rm qb}(\hat{b}^\dagger\hat{\sigma}_- + \hat{b}\hat{\sigma}_+) + \epsilon_{\rm p}(\hat{a} + \hat{a}^\dagger) + \varepsilon_{\rm d}(\hat{\sigma}_+ + \hat{\sigma}_-),\end{aligned} \tag{S4}$$

where $\Delta_{\rm b} = \omega_{\rm d} - \omega_{\rm b}$, $\Delta_{\rm q} = \omega_{\rm d} - \tilde{\Omega}_{\rm q}$, and $\Delta_{\rm r} = \omega_{\rm p} - \omega_{\rm r} + \chi$. We have defined the readout cavity detuning $\Delta_{\rm r}$ to include the Lamb shift; however, this decision is arbitrary. The density matrix of the system $\rho$ has dynamics described by the master equation

$$\partial_t\rho = -\frac{i}{\hbar}[\hat{\mathcal{H}}'_{\rm rot}, \rho] + \kappa\mathcal{L}[\hat{a}]\rho + \gamma_{\rm b}\mathcal{L}[\hat{b}]\rho + \Gamma_1\mathcal{L}[\hat{\sigma}_-]\rho + \frac{\Gamma_\phi}{2}\mathcal{L}[\hat{\sigma}_{\rm z}]\rho. \tag{S5}$$

Here $\kappa$ is the cavity decay, $\gamma_{\rm b}$ is the phonon decay, $\Gamma_1$ is the qubit population decay, and $\Gamma_\phi$ is the qubit dephasing. We have assumed zero temperature and ignored small corrections to the dissipator due to the Schrieffer-Wolff transformation.

### B. Mean field theory for the steady-state phonon population

We can eliminate the microwave cavity mode in (S5) following the procedure outlined in[2]. The procedure corresponds to a displacement of the cavity mode conditioned on the qubit state, followed by a partial trace of the cavity mode under the assumption of no occupation of the cavity fluctuations. More details will be given in a future paper. The procedure yields the following master

$$\partial_t\varrho = -\frac{i}{\hbar}[\hat{\mathcal{H}}_{\rm eff}, \varrho] + \gamma_{\rm b}\mathcal{L}[\hat{b}]\varrho + \Gamma_1\mathcal{L}[\hat{\sigma}_-]\varrho + \frac{\tilde{\Gamma}_\phi}{2}\mathcal{L}[\hat{\sigma}_{\rm z}]\varrho, \tag{S6}$$

where $\varrho$ is the phonon-qubit density matrix, and $\tilde{\Gamma}_\phi$ is the qubit dephasing including the read-out cavity induced dephasing[2]. The effective Hamiltonian reads

$$\frac{\hat{\mathcal{H}}_{\rm eff}}{\hbar} = -\Delta_{\rm b}\hat{b}^\dagger\hat{b} - \frac{\tilde{\Delta}_{\rm q}}{2}\hat{\sigma}_{\rm z} + g_{\rm qb}\left(\hat{b}\hat{\sigma}_+ + \hat{b}^\dagger\hat{\sigma}_-\right) + \varepsilon_{\rm d}\left(\hat{\sigma}_+ + \hat{\sigma}_-\right). \tag{S7}$$

The qubit detuning $\tilde{\Delta}_q$ also considers the modification due to the read-out cavity.

The total qubit dephasing and the qubit detuning are given by

$$\begin{aligned}\tilde{\Gamma}_\phi &= \Gamma_\phi + \Gamma_{\phi,{\rm cav}}(t),\\ \tilde{\Delta}_{\rm q} &= \Delta_{\rm q} - \omega_{\rm q,cav}(t),\\ \omega_{\rm q,cav}(t)(t) &= 2\chi{\rm Re}[\alpha_{\rm g}(t)\alpha_e^*(t)],\\ \Gamma_{\phi,{\rm cav}}(t) &= 2\chi{\rm Im}[\alpha_{\rm g}(t)\alpha_{\rm e}^*(t)].\end{aligned} \tag{S8}$$

Here, $\alpha_{\rm e(g)}(t)$ are the cavity amplitude if the qubit is in the excited (ground) state, given by

$$\begin{aligned}\partial_t\alpha_{\rm e} &= \left[i(\Delta_{\rm r} - \chi) - \frac{\kappa}{2}\right]\alpha_{\rm e} - i\epsilon_{\rm p},\\ \partial_t\alpha_{\rm g} &= \left[i(\Delta_{\rm c} + \chi) - \frac{\kappa}{2}\right]\alpha_{\rm g} - i\epsilon_{\rm p}.\end{aligned} \tag{S9}$$

Since the cavity decay $\kappa$ is significantly larger than the other decays in the system, we consider for now on that the cavity is always in its steady state, such that the amplitudes $\alpha_{\rm e(g)}$ are given by

$$\begin{aligned}\alpha_{\rm e} &= \frac{i\epsilon_{\rm p}}{i(\Delta_{\rm r} - \chi) - \frac{\kappa}{2}},\\ \alpha_{\rm g} &= \frac{i\epsilon_{\rm p}}{i(\Delta_{\rm r} + \chi) - \frac{\kappa}{2}}.\end{aligned} \tag{S10}$$

From the master equation (S6), we can then obtain the following equations for $\langle\hat{b}\rangle = b$, $\langle\hat{\sigma}_-\rangle = s_-$, $\langle\hat{\sigma}_{\rm z}\rangle = s_{\rm z}$:

$$\begin{aligned}\partial_t b &= \left(i\Delta_{\rm b} - \frac{\gamma_{\rm b}}{2}\right)b(t) - ig_{\rm qb}s_-(t),\\ \partial_t s_- &= \left(i\tilde{\Delta}_{\rm q} - \tilde{\gamma}_2\right)s_-(t) + ig_{\rm qb}b(t)s_{\rm z}(t) + i\varepsilon_{\rm q}s_{\rm z}(t),\\ \partial_t s_{\rm z} &= 2is_-(t)\left(g_{\rm qb}b^*(t) + \varepsilon_{\rm d}\right) - 2is_-^*(t)\left(g_{\rm qb}b(t) + \varepsilon_{\rm d}\right) - \gamma_1(s_{\rm z}(t) + 1),\end{aligned} \tag{S11}$$

which assume the mean field approximations $\langle\hat{b}\hat{\sigma}_{\rm z}\rangle \approx \langle\hat{b}\rangle\langle\hat{\sigma}_{\rm z}\rangle = bs_{\rm z}$ and $\langle\hat{b}^\dagger\hat{\sigma}_-\rangle \approx \langle\hat{b}^\dagger\rangle\langle\hat{\sigma}_-\rangle = b^*s_-$. The steady-state phonon number is given by $\bar{n}_b = |\bar{b}|^2$, where $\bar{b}$ is the steady-state of the phonon amplitude $b(t)$. Such steady-state is given by

$$\bar{n}_{\rm b} = |\bar{b}|^2 = \frac{g_{\rm qb}^2\varepsilon_{\rm d}^2\bar{s}_{\rm z}^2}{\left(\Delta_{\rm b}\tilde{\gamma}_2 + \tilde{\Delta}_{\rm q}\frac{\gamma_{\rm b}}{2}\right)^2 + \left(\frac{\gamma_{\rm b}\tilde{\gamma}_2}{2} - \Delta_{\rm b}\tilde{\Delta}_{\rm q} - g_{\rm qb}^2\bar{s}_{\rm z}\right)^2}, \tag{S12}$$

where the steady-state $\bar{s}_{\rm z}$ reads

$$\bar{s}_{\rm z} = -\frac{\gamma_1}{\gamma_1 + 4\frac{\tilde{\gamma}_2}{\tilde{\Delta}_{\rm q}^2 + \tilde{\gamma}_2^2}|g_{\rm qb}\bar{b} + \varepsilon_{\rm d}|^2}. \tag{S13}$$

The equation for the phonon number (S12) is a non-linear equation for $\bar{b}$ that has to be solved numerically. The results for parameters in correspondence with the experiment are shown in Supplementary Fig. 3, in which we can see a good agreement with the full master equation simulation.

We can further manipulate equation (S14) as

$$\bar{n}_{\rm b} = |\bar{b}|^2 = \frac{g_{\rm qb}^2\varepsilon_{\rm d}^2}{\left(\Delta_{\rm b}\frac{\gamma_{\rm eff}}{2} + \Delta_{\rm eff}\frac{\gamma_{\rm b}}{2}\right)^2 + \left(\frac{\gamma_{\rm b}\gamma_{\rm eff}}{4} - \Delta_{\rm b}\Delta_{\rm eff} + g_{\rm qb}^2\right)^2}, \tag{S14}$$

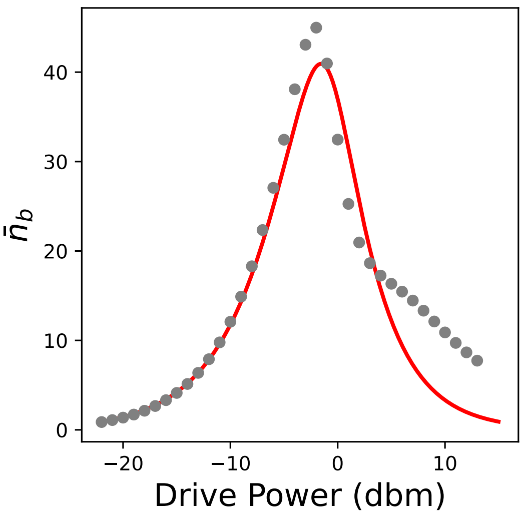


SUPPLEMENTARY FIGURE 3. **Steady-state mean-field phonon number as a function of the qubit drive power**. The red line depicts the result obtained with the mean-field solution of Eq. (S14), and the gray points depict the results from simulations of the full master equation (S5). The parameters used are given in Table I in correspondence with the experimental parameters of the main text.

where we have defined the effective decay $\gamma_{\rm eff}$ and detuning $\Delta_{\rm eff}$ as

$$\begin{aligned} \frac{\gamma_{\rm eff}}{2} &\equiv \frac{\tilde{\gamma}_2}{-\bar{s}_{\rm z}} = \frac{\tilde{\gamma}_2}{\gamma_1}\left(\gamma_1 + 4\frac{\tilde{\gamma}_2}{\tilde{\Delta}_{\rm q}^2+\tilde{\gamma}_2^2}|g_{\rm qb}\bar{b}+\varepsilon_{\rm d}|^2\right), \\ \Delta_{\rm eff} &\equiv \frac{\tilde{\Delta}_{\rm q}}{-\bar{s}_{\rm z}} = \frac{\tilde{\Delta}_{\rm q}}{\gamma_1}\left(\gamma_1 + 4\frac{\tilde{\gamma}_2}{\tilde{\Delta}_{\rm q}^2+\tilde{\gamma}_2^2}|g_{\rm qb}\bar{b}+\varepsilon_{\rm d}|^2\right). \end{aligned} \tag{S15}$$

From such a mean-field perspective, the steady-state of the phonon mode behaves similarly as if it would be coupled to a coherently driven cavity with the nonlinear decays and detunings given by Eq. (S15). We notice that as the power is increased, the effective decay $\gamma_{\rm eff}$ increases, a consequence of the qubit power broadening. Such an effect yields the eventual decoupling between the qubit and the phonon, similar to an inverse Purcell effect. Nevertheless, such a power broadening is not the only physical mechanism that plays a role in the build-up of the phonon steady-state. In fact, both the effective decay and detuning depend on the phonon amplitude $\bar{b}$, a non-linearity that can not be discarded for the experimental parameters. To show this effect, we plot in Fig. 4 the mean-field solution for $n_{\rm b}$ (red curve) and the corresponding curve by setting $g_{\rm qb} = 0$ in Eqs. (S15) (dashed black curve). The latter still captures the inverse Purcell effect due to the qubit power broadening but yields half of the maximum phonon occupancy, indicating the importance of the phonon nonlinearity stemming from the phonon-qubit coupling and the intrinsic two-level nature of the qubit.

### C. Phonon mode linewidth

The semi-classical equations describing the system dynamics (S11) can also be used to obtain information about the phonon mode response in the steady-state, in particular, its effective linewidth. To compute the effective phonon linewidth in the steady-state, we numerically solve Eq. (S11) in time-domain, which gives the time-dependent phonon amplitude $b(t)$. The phonon spectrum is then given by $|b[\omega]|^2$, where $b[\omega]$ is the Fourier transform of $b(t)$. Such a spectrum is a Lorentzian in the frequency domain, with a linewidth that we refer as the Phonon linewidth.

In Supplementary Fig. 5 we shown in (A) the steady-state phonon population as a function of the drive amplitude for the parameters of the experiment and in (B) the corresponding phonon linewdith obtained by fitting the phonon spectrum obtained numerically to a Lorentzian. In (C) and (D) we show two examples of phonon spectrum. We notice that as the drive approaches the upper threshold, the phonon linewidth goes below its intrinsic value, pointing to an amplification of the phonon mode. Such an effect would not be possible only with a decoupling between qubit and phonon, and it is intrinsically related to the non-linear character of the Jaynes-Cummings interaction. Furthermore,

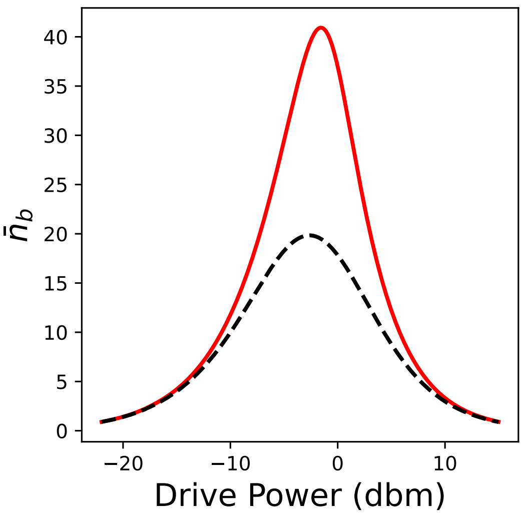


SUPPLEMENTARY FIGURE 4. **Steady-state mean-field phonon number as a function of the qubit drive power**. The red line depicts the result obtained with the mean-field solution of Eq. (S14), while the black dashed line depicts the result obtaining by discarding the phonon nonlinearity in eqs. (S15) The parameters used are given in Table I in correspondence with the experimental parameters of the main text.

the reduction of the effect linewidth is what enables the build-up of such a large steady-state phonon population.

### D. Qubit Two-Tone Spectroscopy

Following the arguments presented in[3], ignoring the qubit-phonon coupling, in the dispersive regime, $\arg[\langle \hat{a}(t)\rangle]$ is directly related to the qubit population $\langle \hat{\sigma}_z(t)\rangle$. By recording the phase of the readout resonator, one can then obtain the qubit absorption spectrum

$$S(\omega) = \frac{1}{2\pi}\int_{-\infty}^{\infty} dt e^{i\omega t}\langle \hat{\sigma}_-(t)\hat{\sigma}_+(0)\rangle_s, \tag{S16}$$

where $\langle\cdot\rangle_s$ indicates that the expectation value is taken in the steady state.

It was shown that for a qubit-cavity system, the qubit absorption spectrum is given by[3]

$$S(\omega) = \frac{1}{\pi}\sum_{j=0}^{\infty}\frac{1}{j!}\mathrm{Re}\left(\frac{(-A)^j e^A}{\Gamma_{\mathrm{q}}^{(j)}/2 - i(\omega - \Omega_{\mathrm{q}}^{(j)})}\right) \equiv \sum_{j=0}^{\infty} S_j(\omega), \tag{S17}$$

with

$$\begin{aligned}
A &= D_{ss}\left(\frac{\kappa/2 - 2i\chi}{\kappa/2 + 2i\chi}\right),\\
B &= \chi(\bar{n}_e + \bar{n}_g - D_{ss}),\\
D_{ss} &= \frac{2\chi^2(\bar{n}_e + \bar{n}_g)}{(\kappa/2)^2 + 2\chi^2},\\
\bar{n}_e &= \frac{\bar{n}_g(\kappa/2)^2}{(\kappa/2)^2 + (2\chi)^2},\\
\Gamma_{\mathrm{q}}^{(j)} &= 2\gamma_{\mathrm{q}} + \kappa(j + D_{ss}),\\
\omega_{\mathrm{q}}^{(j)} &= \tilde{\omega}_q + B + 2j\chi.
\end{aligned} \tag{S18}$$

In the above equations, $\omega_{\mathrm{q}}^{(j)}$ and $\Gamma_{\mathrm{q}}^{(j)}$ are the frequency and linewidth of the qubit with the readout resonator in the state $|j\rangle$, respectively. The intrinsic qubit linewidth, with the readout resonator in its ground state, is given by

$\gamma_q = \Gamma_1/2 + \Gamma_\phi$, where $\Gamma_1$ is the longitudinal relaxation rate, and $\Gamma_\phi$ is the pure dephasing rate. The qubit frequency $\omega_q^{(0)}$ is ac Stark shifted by $B$ from its intrinsic value $\omega_q$. We have also assumed that the readout drive is on resonance with the readout resonator, i.e. $\Delta_r = 0$, and the readout resonator has a full-width half-maximum linewidth $\kappa$. In the limit $\chi \sim \kappa$ and with $\Delta_r = 0$, the components $S_j(\omega)$ have non-Lorentzian lineshapes and can even be negative. The sum of these individual components can result in an asymmetry of the qubit spectrum, as seen in Supplementary Fig. 6(a).

We perform spectroscopy of the qubit by monitoring the transmission coefficient $S_{21}$ of the readout resonator as a function of the qubit drive frequency $\omega_d$. The probe tone was fixed at the Stark-shifted readout resonator frequency with the qubit in its ground state $\omega_c^g/2\pi = 4.91$ GHz, such that $\Delta_r = 0$ and held at a constant power $\mathcal{P}_d = -25$ dBm, set at room temperature. The probe line has a total of 108 dB of attenuation, ensuring the average number of photons in the probe mode on average is much less than one.

To fit the measured spectrum, we use the expression,

$$|S_{21}| = \mathcal{A} \sum_{j=0}^{10} S_j(\omega) + \mathcal{C}, \tag{S19}$$

where $\mathcal{A}$ is a conversation factor between $S_j(\omega)$ and $|S_{21}|$ and $\mathcal{C}$ is a constant offset of the spectrum. The value of the Fock basis was truncated to $j = 10$, and the linewidth of the readout resonator was independently determined and fixed; see Table. I. The fitting parameters include the intrinsic qubit frequency $\omega_q$, the power broadened qubit linewidth $\Gamma_q(\mathcal{P}_d)$, where $\mathcal{P}_d$ is the qubit drive power, the qubit dispersive shift $\chi$, the probe mode occupancy with the qubit in its ground state $\bar{n}_g$, and conversion factor $\mathcal{A}$, and the constant offset $\mathcal{C}$.

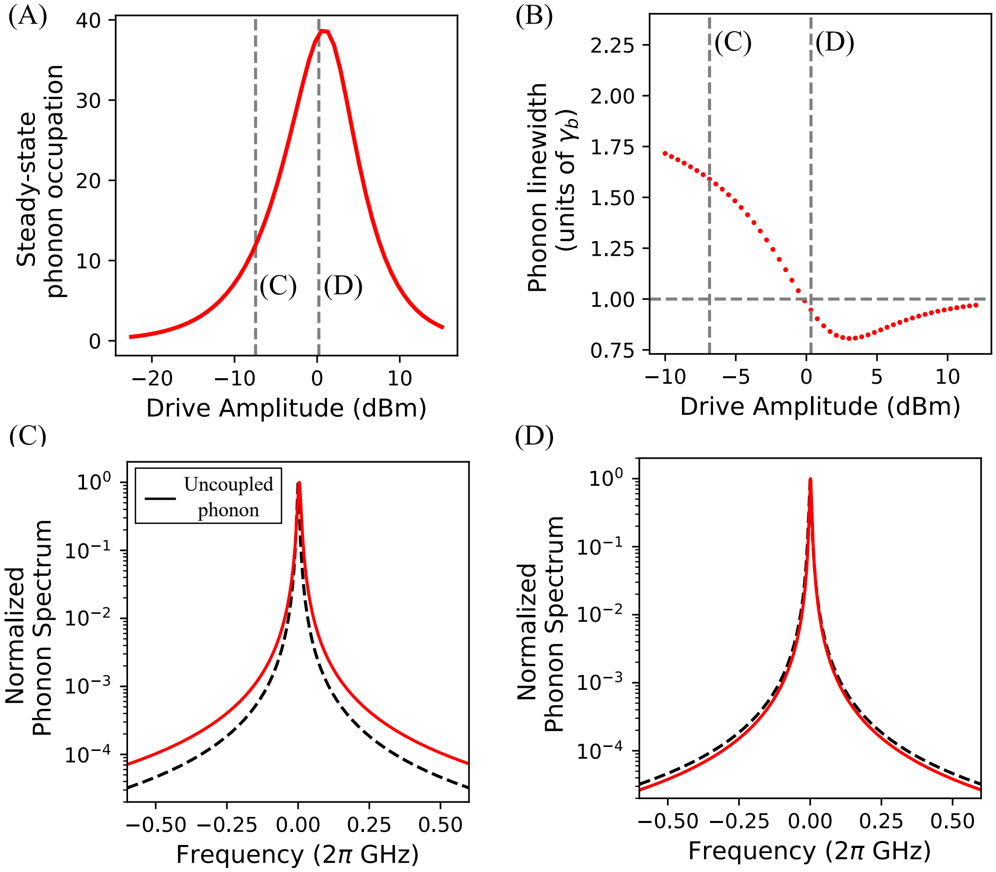


SUPPLEMENTARY FIGURE 5. **Linewidth narrowing of the HBAR mode** (A) Steady-state phonon occupation for the system in the manuscript. (B) Effective phonon linewidth extracted from a Lorentzian fit of the phonon spectrum. (C,D) phonon spectra at two representative drive amplitudes showing the linewidth narrowing with power, the black dashed line depicts the spectrum for a phonon mode uncoupled from the two-level system. Parameters in correspondence with the experiment.

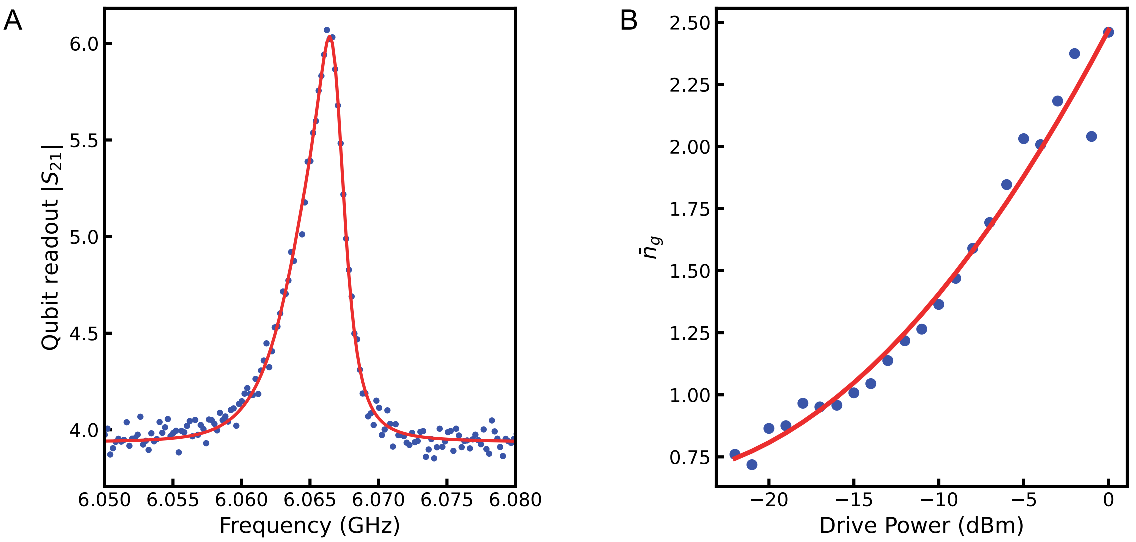


SUPPLEMENTARY FIGURE 6. **Two-tone qubit spectroscopy measurements.** (A) Experimental two-tone qubit spectrum (blue) and the qubit spectrum determined from a fit to Eq. S19 (red). The qubit drive power at room temperature was set to -20.0 dBm. (B) Extracted value of the readout resonator photon population as a function of qubit drive power.

An example spectrum and its fit are shown in Supplementary Fig. 6(a). First, the value of the dispersive shift was determined to be $\chi/2\pi = -1.2 \pm 0.2$ MHz, which agreed with our designed value. This value was then fixed, and the data was fit for all qubit drive powers to determine the remaining values. We found that the ground state readout photon population depended on the qubit drive power. This is likely due to the heating of the silicon substrate because the qubit drive tone was applied via the readout resonator. The value of $\bar{n}_\mathrm{g}(\mathcal{P}_\mathrm{d})$ is shown in Supplementary Fig. 6(b). The zero power qubit linewidth was also extracted by extrapolating the measured power-broadened qubit linewidth to zero power and was determined to be $\gamma_\mathrm{q}(0)/2\pi = 0.420 \pm 0.04$ MHz, which agrees with our $\gamma_\mathrm{q}/2\pi = \Gamma_1/2 + \Gamma_\phi \geq 1/2T_1$ limit determined using a time-domain measurement with $T_1 = 180 \pm 30$ ns. The comparison between our spectroscopy and time domain measurements indicates that our qubit decay is dominated by decoherence, and therefore we have ignored intrinsic dephasing in our model since qubit dephasing will be dominated by power-induced dephasing induced by the qubit drive tone.

## E. Master Equation Simulations

### Qubit Spectroscopy

We simulate the dynamics of the master equation Eq. S5 using the Python package Qutip[4]. We first compare the measured qubit spectrum without the phonon mode to the Qutip steady-state simulations. To begin, we must consider the non-zero photon population of the readout resonator. The finite population results in an asymmetry in the qubit spectrum, as well as additional measurement-induced dephasing. To include the finite readout population in our simulation, the readout drive $\epsilon_\mathrm{p}$ was set such that the average population $\langle \hat{a}^\dagger \hat{a} \rangle = \bar{n}_\mathrm{g}(\mathcal{P}_\mathrm{d})$. This ensured that for each qubit drive power, the readout resonator had the appropriate number of steady-state photons.

To account for qubit power-broadening, the qubit drive power in the simulation had to be calibrated. As stated above, $\varepsilon_\mathrm{d} = \frac{g_\mathrm{qc}\epsilon_\mathrm{d}}{\omega_\mathrm{r} - \Omega_\mathrm{q}}$, where $\epsilon_\mathrm{d} = \sqrt{\kappa_\mathrm{ext}}\sqrt{\mathcal{P}_\mathrm{d}/\hbar\omega_\mathrm{d}}$, $\kappa_\mathrm{ext}$ is the external coupling rate to the readout resonator, and $\mathcal{P}_\mathrm{d}$ is the drive power in Watts at the coupling port of the readout resonator. Therefore, the drive coefficient $\varepsilon_\mathrm{d}$ can be written in the form

$$\varepsilon_\mathrm{d} = \sqrt{10^{(\mathcal{P}_\mathrm{RT} + \delta)/10}} \tag{S20}$$

where $\delta$ calibrates the room-temperature power to the corresponding value of $\mathcal{P}_\mathrm{d}$ accounting for all losses and multiplicative factors. The value of $\delta$ was determined by matching the simulation to the power-broadened qubit spectrum at multiple qubit drive powers. A set of qubit spectra is shown in Supplementary Fig. 7 where it can be seen that the master equation simulation is in excellent agreement capturing the qubit asymmetry at low power, and the

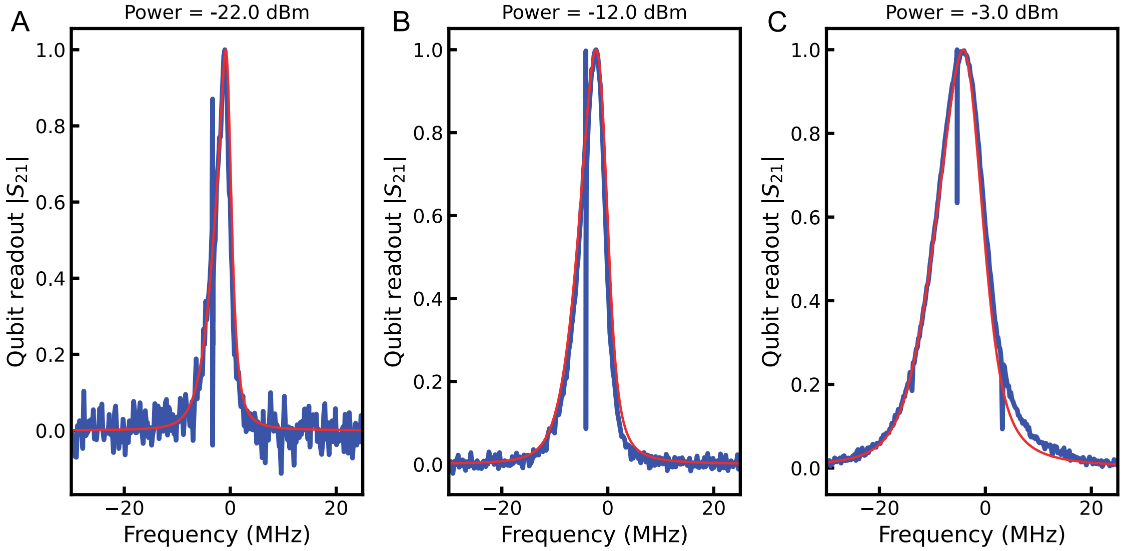


SUPPLEMENTARY FIGURE 7. **Two-tone qubit spectroscopy measurements.** (A) Experimental two-tone qubit spectrum (blue) overlapped with the qubit spectrum determined from the master equation simulation (red). The frequency is defined as the detuning from the bare qubit frequency. The qubit drive power at room temperature was set to -12.0 dBm. (B) Qubit spectrum and master equation simulation for a qubit drive power of -6.0 dBm. (C) Qubit spectrum and master equation simulation for a qubit drive power of -3.0 dBm. The feature at $\sim$ 0 MHz is an additional HBAR mode, one free-spectral range from the HBAR mode of interest for this work. The only parameter that was varied within the simulation was the room-temperature value of the qubit drive.

power-broadened qubit spectrum at higher drive powers. It should be noted the measured transmission signal $S_{21}$ is proportional to the qubit population $\langle\hat{\sigma}_z\rangle$ only if the appropriate signal quadrature is measured. Here, this was determined by calculating the rotation angle that minimized the signal in the out-of-phase quadrature. To confirm, we compared our single-trace two-tone data with a direct measurement of the readout resonator frequency at several qubit drive powers. At the highest drive powers, we observed a slight deviation resulting from signal mixing into the out-of-phase quadrature. However, this was at the drive powers above the self-quenching threshold and resulted in a slight mismatch between our experimental and simulated two-tone traces.

## Gated Ringdown

Following the calibration of the qubit spectrum, the phonon mode was included in the master equation simulation. The value of the coupling rate $g_{\rm qb}$ and the phonon linewidth $\gamma_{\rm b}$ were determined by performing a fit to the time domain gated two-tone measurements. The fit was determined by fitting both the ringdown (as shown in the main text) and also the ring-up of the qubit. Here, the device was allowed to thermalize before the gated-ringdown measurement, weakly probing the readout resonator continually. At the time $t = 0$, the qubit drive is switched on for $175\mu$s, allowing the phonon moded to ring-up. At $t = 175\mu$s, the qubit drive is switched off, and the phonon ringdown is observed, as described in the main text. The best-fit values were compared at multiple drive powers; see Supplementary Fig. 8(a). We extracted a value of the qubit phonon coupling of $g_{\rm qb}/2\pi = 162$ kHz and a phonon linewidth of $\gamma_{\rm b}/2\pi = 6.81$ kHz. From this set of simulations, the steady-state phonon population and phonon statistics can be estimated for multiple drive powers. We observe a good agreement between the numerical simulation and the ring-up data and an excellent agreement between the ringdown data for all powers. Deviations in the ring-up simulations likely result from higher-order nonlinearities we are not considering in our model.

The master equation simulations directly calculate the time dynamics of $\langle\hat{\sigma}_+\hat{\sigma}_-\rangle$, this value has been scaled by the same constant factor for all powers to give a direct comparison to our two-tone measurement. Since we expect the variation in the transmission coefficient measured in the two-tone measurement to be proportional to $\langle\hat{\sigma}_+\hat{\sigma}_-\rangle$, see Eq. S3 and Ref.[3]. From our simulation, we calculate the phonon population and second-order correlation function for multiple drive powers, shown in Supplementary Fig. 8(b,c). For low drive powers, the phonon statistics are described by a coherent state, $g^{(2)}(0) \approx 1.0$. However, as discussed in the main text, the phonon amplitude exhibits self-quenching above a given upper-threshold power. This can be seen in both the phonon population, as a rapid decrease

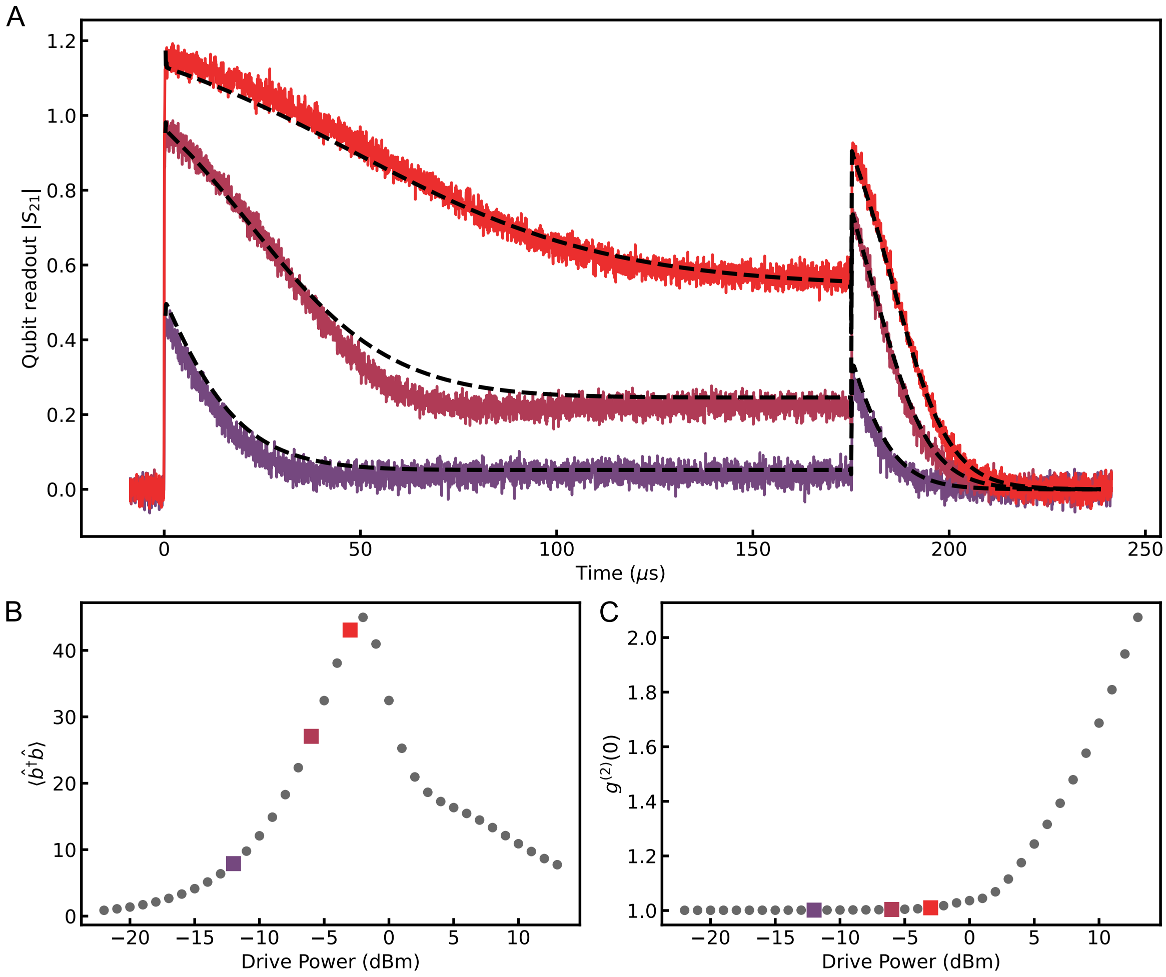


SUPPLEMENTARY FIGURE 8. **Gated two-tone qubit spectroscopy.** (A) Experimental gated two-tone measurements for qubit drive powers -12.0, -6.0, -3.0 dBm. The master equation simulations at the same drive power are plotted as dashed black lines. The simulation has an excellent agreement for the ringdown data for all powers and a slight deviation from the ring-up data. This could be attributed to higher-order non-linearities we are not considering within our simple master equation model. However, the agreement between the experiment and simulation is good for all powers. (B) Simulated phonon state population $\langle \hat{b}^\dagger \hat{b} \rangle$ as a function of qubit drive power. (C) Simulated second-order phonon correlations $g^{(2)}(0)$ as a function of qubit drive power.

in the populations, and in the phonon statistics as $g^{(2)}(0) > 1.0$ for drive powers above the self-quenching threshold.

## III. SUPPLEMENTARY NOTE 3: SIMPLIFIED MODEL

### A. Considerations of Qubit Anharmonicity

We modeled our system using a two-level approximation; however, under strong drives, it is important to consider the higher levels of the transmon qubit. We can instead model the qubit as a Kerr oscillator with a large anharmonicity $\alpha$. Therefore, we can re-write the Hamiltonian in the form

$$\frac{\hat{\mathcal{H}}_{\mathrm{mod}}}{\hbar} = -\Delta_{\mathrm{q}}\hat{c}^\dagger\hat{c} + \frac{\alpha}{2}\hat{c}^\dagger\hat{c}^\dagger\hat{c}\hat{c} - \Delta_{\mathrm{b}}\hat{b}^\dagger\hat{b} + g_{\mathrm{qb}}(\hat{b}^\dagger\hat{c} + \hat{b}\hat{c}^\dagger) + \varepsilon_{\mathrm{d}}(\hat{c}^\dagger + \hat{c}). \quad \text{(S21)}$$

Where we have replaced the two-level raising and lowering operators with bosonic raising and lowering operators $\hat{c}^{(\dagger)}$. If we calculate the number of steady-state phonons comparing the Kerr oscillator model to the two-level system model,

SUPPLEMENTARY TABLE I. Symbols and parameters

| Parameter | Symbol |
|---|---|
| Microwave mode frequency | $\omega_c = 2\pi \times 4.910$ GHz |
| Microwave mode decay | $\kappa = 2\pi \times 2.897$ MHz |
| Phonon mode frequency | $\omega_b = 2\pi \times 6.064$ GHz |
| Phonon mode decay | $\gamma_b = 2\pi \times 6.81$ kHz |
| Qubit frequency | $\Omega_q = 2\pi \times 6.067$ GHz |
| Qubit energy relaxation rate | $\Gamma_1 = 2\pi \times 0.840$ MHz |
| Qubit phase relaxation rate | $\Gamma_\phi < 2\pi \times 0.08$ MHz |
| Qubit anharmonicity | $\alpha = -2\pi \times 260.0$ MHz |
| Qubit-phonon coupling | $g_{qb} = 2\pi \times 162$ kHz |
| Dispersive cavity-qubit coupling | $\chi = -2\pi \times 1.2$ MHz |
| Lamb-shifted qubit frequency | $\tilde{\Omega}_q = \Omega_q - \chi$ |
| Qubit drive frequency | $\omega_d$, varied around $\Omega_q$ |
| Cavity probe frequency | $\omega_p$, set at the Stark-shifted cavity frequency |

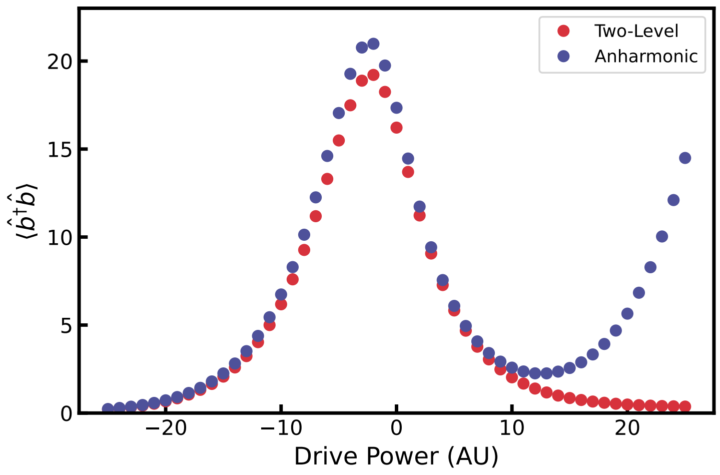


SUPPLEMENTARY FIGURE 9. **Two-Level versus Anharmonic Oscillator Simulations.** The simulated phonon population when considering the transmon qubit as a two-level system versus considering three levels of the Kerr oscillator.

we find that at powers below and near the upper threshold, the population is slightly larger in the Kerr oscillator model; see Supplementary Fig. 9. There exists a discrepancy at high drive powers; however, this was not observed within the experiment since we likely could not apply a large enough drive without heating the sample. Thus, we are confident that modeling our experiment as a two-level system is accurate, especially for calculating the upper threshold. Moreover, this was required for numerical efficiency. The two-level system simulation in Supplementary Fig. 9 ran for approximately 20 minutes, whereas considering only three levels of the Kerr oscillator increased the simulation time to approximately 10 hours.

For numerical efficiency, we have constructed a simplified model where we have removed the coupling to the readout resonator. Studying Eq. S3, the readout resonator broadens the qubit and causes a constant frequency offset due to steady-state photons in the cavity. The Hamiltonian of our simplified model is given by

$$\frac{\hat{\mathcal{H}}_{\mathrm{mod}}}{\hbar} = -\frac{\Delta_q}{2}\hat{\sigma}_z - \Delta_b \hat{b}^\dagger \hat{b} + g_{qb}(\hat{b}^\dagger \hat{\sigma}_- + \hat{b}\hat{\sigma}_+) + \varepsilon_d(\hat{\sigma}_+ + \hat{\sigma}_-), \tag{S22}$$

and we choose to have the qubit and phonon resonant for the simulation. Moreover, we have increased the intrinsic qubit linewidth $\Gamma_q/2\pi = 1.5$ MHz to account for the lack of readout resonator broadening. We also increased the phonon linewidth $\gamma_b/2\pi = 25$ kHz to reduce the size of the Hilbert space for numerical efficiency.

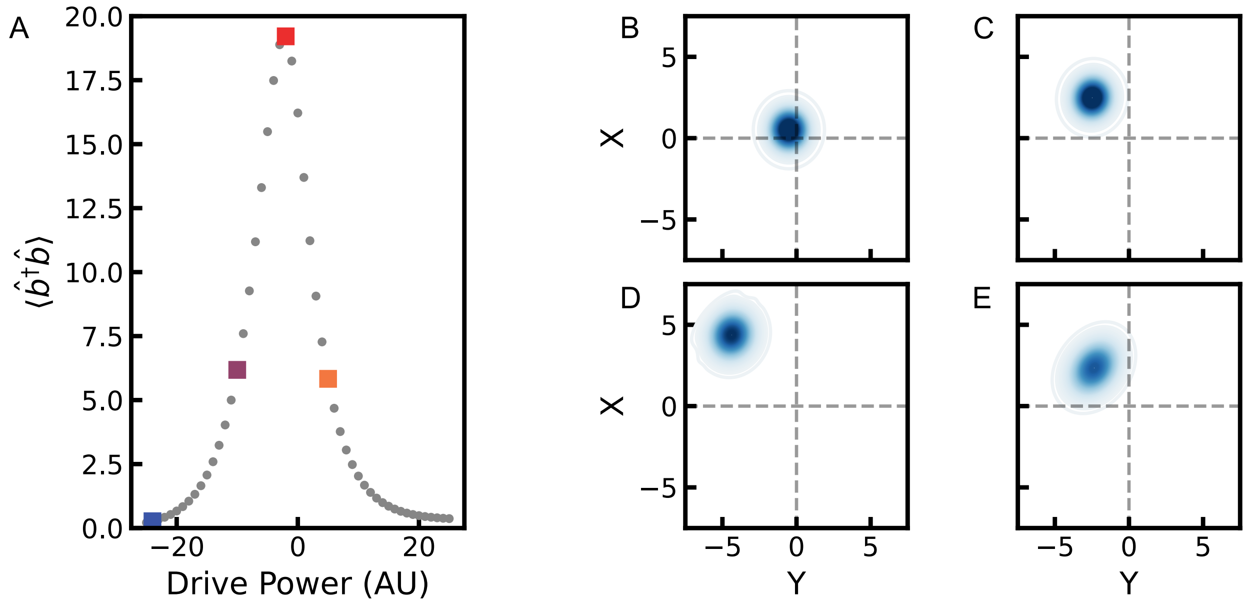


SUPPLEMENTARY FIGURE 10. **Phonon Phase Seeding.** (A) Phonon population as a function of qubit drive power. Colored points correspond to the Wigner plots (B-E). (B-E) Wigner distribution plots corresponding to the points in (A). The phase of the coherent state is set by the drive phase; here, the drive phase was $\pi/4$. The plots are shown starting from the lowest power (B) to the largest power (E).

### B. Phonon Phase Coherence

First, we aim to investigate if the drive tone seeds the phase of the phonon mode. In the model described in Ref.[5] the phase of the phonon mode is random and results in a distribution in the IQ plane. However, if we examine the phase space distribution of the phonon mode in our model, we find that the phase of the drive tone instead sets the phase; see Supplementary Fig. 10. This results from the weak hybridization between the qubit and phonon mode. When the drive is switched on, the weak hybridization enables the seeding of the phase of the phonon mode. This is followed by the stimulated emission of phonons, which produces a large coherent state with a set phase. The ability to set the phase of the phonon mode is important for applications such as the generation of Schrödinger cat states.

### C. Phonon Mode Anharmonicity

Finally, the coupling between the qubit and phonon mode raises the question of how much anharmonicity is inherited by the phonon mode. In the strong coupling limit, the phonon mode will inherit half of the qubit anharmonicity and, thus, would be unable to populate the phonon mode since its Hilbert space is truncated to a single excitation. From the transparency window measurement, we observe little frequency shift of the phonon mode, suggesting the mode is well approximated as a harmonic oscillator and *not* a Duffing oscillator. However, again, we can utilize our simplified model to explore the inherited anharmonicity by directly driving the phonon mode in the simulation. We find that at a drive strength that generates, on average, fifty phonons, similar to what was observed in the experiment, the phonon mode has a negligible frequency shift. Suggesting that in the weak hybridization limit, the phonon mode can be treated to a high degree as a linear harmonic oscillator.

[1]W. J. M. Franse, C. A. Potts, V. A. S. V. Bittencourt, A. Metelmann, and G. A. Steele, "High-coherence quantum acoustics with planar superconducting qubits," (2024), arXiv:2410.10272 [quant-ph].

[2]J. Gambetta, A. Blais, M. Boissonneault, A. A. Houck, D. I. Schuster, and S. M. Girvin, "Quantum trajectory approach to circuit qed: Quantum jumps and the zeno effect," Phys. Rev. A **77**, 012112 (2008).

[3]J. Gambetta, A. Blais, D. I. Schuster, A. Wallraff, L. Frunzio, J. Majer, M. H. Devoret, S. M. Girvin, and R. J. Schoelkopf, "Qubit-photon interactions in a cavity: Measurement-induced dephasing and number splitting," Physical Review A **74**, 042318 (2006).

[4]J. R. Johansson, P. D. Nation, and F. Nori, "Qutip: An open-source python framework for the dynamics of open quantum systems," Computer Physics Communications **183**, 1760–1772 (2012).

[5]S. Ashhab, J. Johansson, A. Zagoskin, and F. Nori, "Single-artificial-atom lasing using a voltage-biased superconducting charge qubit," New Journal of Physics **11**, 023030 (2009)
